# Effect of clinical decision support for severe hypercholesterolemia on low-density lipoprotein cholesterol levels

**DOI:** 10.1038/s41746-024-01069-w

**Published:** 2024-03-18

**Authors:** Hana Bangash, Seyedmohammad Saadatagah, Mohammadreza Naderian, Marwan E. Hamed, Lubna Alhalabi, Alborz Sherafati, Joseph Sutton, Omar Elsekaily, Ali Mir, Justin H. Gundelach, Daniel Gibbons, Paul Johnsen, Christina M. Wood-Wentz, Carin Y. Smith, Pedro J. Caraballo, Kent R. Bailey, Iftikhar J. Kullo

**Affiliations:** 1 Department of Cardiovascular Medicine, Mayo Clinic, Rochester, MN USA; 2 Department of Information Technology, Mayo Clinic, Rochester, MN USA; 3 Department of Quantitative Health Sciences, Mayo Clinic, Rochester, MN USA; 4 Department of General Internal Medicine, Mayo Clinic, Rochester, MN USA; 5 Gonda Vascular Center, Mayo Clinic, Rochester, MN USA

**Keywords:** Translational research, Dyslipidaemias, Preventive medicine, Genetic testing

## Abstract

Severe hypercholesterolemia/possible familial hypercholesterolemia (FH) is relatively common but underdiagnosed and undertreated. We investigated whether implementing clinical decision support (CDS) was associated with lower low-density lipoprotein cholesterol (LDL-C) in patients with severe hypercholesterolemia/possible FH (LDL-C ≥ 190 mg/dL). As part of a pre-post implementation study, a CDS alert was deployed in the electronic health record (EHR) in a large health system comprising 3 main sites, 16 hospitals and 53 clinics. Data were collected for 3 months before (‘silent mode’) and after (‘active mode’) its implementation. Clinicians were only able to view the alert in the EHR during active mode. We matched individuals 1:1 in both modes, based on age, sex, and baseline lipid lowering therapy (LLT). The primary outcome was difference in LDL-C between the two groups and the secondary outcome was initiation/intensification of LLT after alert trigger. We identified 800 matched patients in each mode (mean ± SD age 56.1 ± 11.8 y vs. 55.9 ± 11.8 y; 36.0% male in both groups; mean ± SD initial LDL-C 211.3 ± 27.4 mg/dL vs. 209.8 ± 23.9 mg/dL; 11.2% on LLT at baseline in each group). LDL-C levels were 6.6 mg/dL lower (95% CI, −10.7 to −2.5; *P* = 0.002) in active vs. silent mode. The odds of high-intensity statin use (OR, 1.78; 95% CI, 1.41–2.23; *P* < 0.001) and LLT initiation/intensification (OR, 1.30, 95% CI, 1.06–1.58, *P* = 0.01) were higher in active vs. silent mode. Implementation of a CDS was associated with lowering of LDL-C levels in patients with severe hypercholesterolemia/possible FH, likely due to higher rates of clinician led LLT initiation/intensification.

## Introduction

Severe hypercholesterolemia/possible familial hypercholesterolemia (FH), defined as low-density lipoprotein cholesterol (LDL-C) ≥ 190 mg/dL, is a relatively prevalent disorder that increases risk of coronary heart disease (CHD)^1–5^. The associated increased morbidity and mortality could be reduced by early detection and initiation of lipid lowering therapy (LLT) including statins^6–10^. Despite a recommendation by the American Heart Association/American College of Cardiology (AHA/ACC) to initiate high-intensity statin therapy for individuals with severe hypercholesterolemia/possible FH, treatment remains suboptimal^1^. We previously demonstrated high prevalence and undertreatment of severe hypercholesterolemia/possible FH in Olmsted County, Minnesota^1^. In the period between 2004–2015, 1 of 11 adults had at least one LDL-C level of ≥ 190 mg/dL and guideline-directed therapeutic targets for LDL-C in the primary and secondary prevention settings were achieved in less than half the individuals^1^. Similar results were noted in the United States CASCADE-FH Registry; of 1295 patients with FH or possible FH on LLT, only 25% had LDL-C levels < 100 mg/dL^6,11^.

To address the underdiagnosis and undertreatment of FH, we developed a suite of digital tools including an electronic algorithm to identify possible FH cases in the electronic health record (EHR)^12^, a clinical decision support (CDS) tool to alert clinicians regarding next steps in management^13–15^, an FH Conversation Aid to facilitate shared decision making regarding LLT for FH^16^, and a Web App to facilitate sharing of genetic test results in families with FH^17^. The CDS for FH was developed based on input from key stakeholders, formatted as an in-basket message alert, and then deployed in silent mode and active mode for 3 months each, to collect data by which to compare outcomes. Using a pre-post implementation study design, we investigated whether the CDS would lead to lower LDL-C in patients with levels ≥ 190 mg/dL.

## Results

### Baseline characteristics

**Fig. 1:**
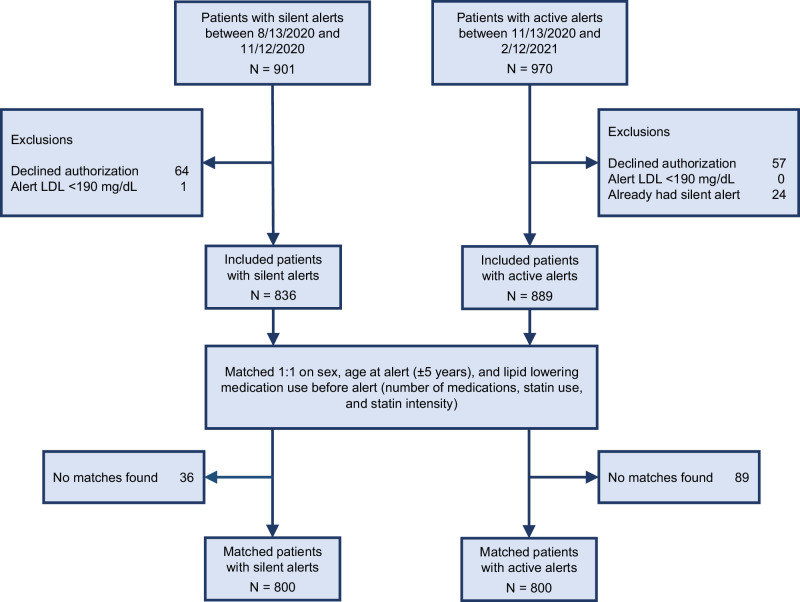
Patient selection and matching in the pre-post implementation groups. CDS clinical decision support, EHR electronic health record, LDL-C low-density lipoprotein cholesterol, LLT lipid lowering therapy.

The CDS alert triggered for 901 patients in silent mode and for 970 patients in active mode (Fig. 1). Of these, 836 met study inclusion criteria in the silent mode group and 889 in the active mode group; in 24 patients the alert triggered in both silent mode and active mode and these patients were excluded from the active mode period. After matching, a total of 1600 patients were identified, 800 in the silent mode group and 800 in the active mode group. Nearly half of the patients (374 in silent mode, and 384 in active mode) triggered the alert in the Mayo Health System clinics serving primarily rural areas across Minnesota and Wisconsin, and the remaining patients were from medical centers in Rochester, Jacksonville, and Phoenix/Scottsdale (serving primarily urban areas) (Supplementary Table 1).

Patient baseline demographic and clinical characteristics at the time of alert trigger are shown in Table 1. As patients in silent mode and active mode were matched, both groups had similar age (mean ± SD 56.1 ± 11.8 y vs. 55.9 ± 11.8 y) with 36.0% males in both modes. The baseline LDL-C level in both groups was similar (mean ± SD 211.3 ± 27.4 mg/dL vs. 209.8 ± 23.9 mg/dL; *P* = 0.26). Self-reported race was similar in both groups and most patients identified as White (90.0% in silent mode vs. 91.9% in active mode; *P* = 0.81). The proportions of those with hypertension, obesity, and who smoked were also similar between the two groups (Table 1). The use of LLT prior to the alert was similar in the silent and active mode groups due to matching. Only 11.2% of individuals in each group were on LLT within 30 days prior to alert trigger, as shown in Table 1.

**Table 1:** Baseline characteristics of patients

Characteristic	*N* (%)		*P* value^a^
	Silent mode (*n* = 800)	Active mode (*n* = 800)	
Age, mean ± SD, y^b^	56.1 ± 11.8	55.9 ± 11.8	0.85^b^
Female^b^	512 (64.0%)	512 (64.0%)	NA^b^
Baseline LDL-C, mean ± SD, mg/dL	211.3 ± 27.4	209.8 ± 23.9	0.26
Self-reported race			0.81
American Indian or Alaskan Native	4 (0.5%)	3 (0.4%)	
Asian	24 (3.0%)	17 (2.1%)	
Black or African American	33 (4.1%)	29 (3.6%)	
White	720 (90.0%)	735 (91.9%)	
Other^c^	9 (1.1%)	9 (1.1%)	
Unknown	10 (1.3%)	7 (0.9%)	
Self-reported ethnicity			0.87
Hispanic or Latino	37 (4.6%)	34 (4.3%)	
Not Hispanic or Latino	748 (93.5%)	753 (94.1%)	
Unknown	15 (1.9%)	13 (1.6%)	
Coronary heart disease	48 (6.0%)	25 (3.1%)	0.006
Diabetes	102 (12.8%)	74 (9.3%)	0.03
Hypertension	308 (38.5%)	289 (36.1%)	0.33
Smoking status			0.66
Current	87 (10.9%)	92 (11.5%)	
Former	217 (27.1%)	230 (28.8%)	
None	485 (60.6%)	471 (58.9%)	
Unknown	11 (1.4%)	7 (0.9%)	
Body mass index			0.93
<25 kg/m^2^	153 (19.1%)	161 (20.1%)	
25.0 – 29.9 kg/m^2^	310 (38.8%)	303 (37.9%)	
≥30 kg/m^2^	326 (40.8%)	323 (40.4%)	
Unknown	11 (1.4%)	13 (1.6%)	
Department of practice			<0.001
Primary care	622 (77.8%)	674 (84.3%)	
Subspeciality clinic	178 (22.3%)	125 (15.6%)	
Unknown	0 (0.0%)	1 (0.1%)	
Clinician category			0.77
MD/DO	598 (74.8%)	592 (74.0%)	
Non-MD/DO (NP or PA)	202 (25.3%)	207 (25.9%)	
Unknown	0 (0.0%)	1 (0.1%)	
Location of medical facility			0.45
Academic medical center (urban)^e^	398 (49.8%)	382 (47.8%)	
Health system clinic (rural)^f^	402 (50.3%)	417 (52.1%)	
Unknown	0 (0.0%)	1 (0.1%)	
LLT regimen before the alert^b^			NA^b^
No LLT	710 (88.8%)	710 (88.8%)	
Single medication class^d^	89 (11.1%)	89 (11.1%)	
Two medication classes^d^	1 (0.1%)	1 (0.1%)	
Statin use before the alert^b^	80 (10.0%)	80 (10.0%)	NA^b^
Statin intensity^b^			NA^b^
No statin use	720 (90.0%)	720 (90.0%)	
Low	6 (0.8%)	6 (0.8%)	
Moderate	44 (5.5%)	44 (5.5%)	
High	30 (3.8%)	30 (3.8%)	
Non statin use before the alert^b^	11 (1.4%)	11 (1.4%)	NA^b^

*DO* doctor of osteopathic medicine, *LDL-C* low-density lipoprotein cholesterol, *LLT* lipid lowering therapy, *MD* doctor of medicine, *NA* not applicable, *NP* nurse practitioner, *PA* physician assistant, *SD* standard deviation.

^a^*P* values were calculated using *t*-tests, chi-square tests, or Fisher’s exact test.

^b^Characteristics used in matching participants in silent and active mode groups.

^c^Other race includes Native Hawaiian, Pacific Islander, and mixed race.

^d^Medication classes include statins, bile acid sequestrants, ezetimibe, fibrates, monoclonal antibodies, niacin, and other non-statins.

^e^Academic medical centers located in 3 urban areas: Rochester, Minnesota; Phoenix and Scottsdale, Arizona; and Jacksonville, Florida.

^f^Health system clinics located in several small towns / rural communities in Minnesota and Wisconsin.

### Outcomes

**Fig. 2:**
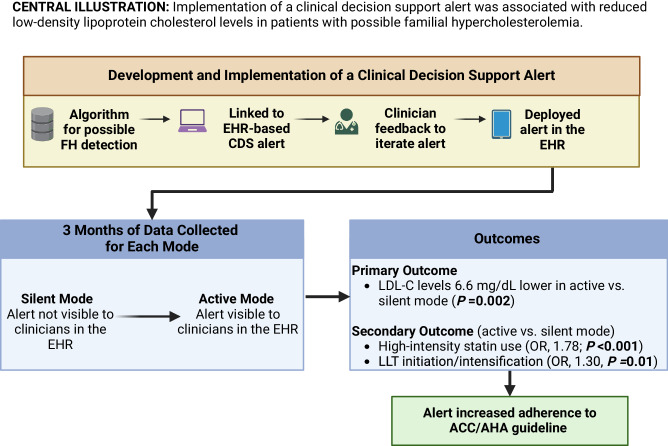
Implementation of a clinical decision support alert was associated with lowering of low-density lipoprotein cholesterol levels in patients with possible familial hypercholesterolemia. FH familial hypercholesterolemia, EHR electronic health record, CDS clinical decision support, LDL-C low-density lipoprotein cholesterol, LLT lipid lowering therapy, ACC/AHA American College of Cardiology/American Heart Association.

A total of 415 (51.9%) patients in the silent mode group and 484 (60.5%; *P* < 0.001) patients in the active mode group had LDL-C measured in the 1–12 months after alert trigger. LDL-C levels were significantly lower (estimate, −6.6; 95% CI, −10.7 to −2.5; *P* = 0.002) in the active mode patients (Table 2, Fig. 2). The results were similar in sensitivity analysis using the latest LDL-C. More patients in the active mode group were on statins (59.6% vs. 53.8%; *P* = 0.02) and high-intensity statins (32.5 vs. 22.6%; *P* < 0.001) than in the silent mode group (Supplementary Table 2). The odds of any statin use (OR, 1.32; 95% CI, 1.08–1.61; *P* = 0.007), high intensity statin use (OR, 1.78; 95% CI,1.41–2.23; *P* < 0.001) and of initiation or intensification of LLT (OR, 1.30; 95% CI, 1.06–1.58; *P* = 0.01) were greater in active mode compared to silent mode (Table 2). Among patients with LLT initiation or intensification after the CDS alert, the median time from the changes in LLT to subsequent LDL-C testing was similar between the silent mode and the active mode groups (median [interquartile range] 96 [85–155] days in the silent mode vs 91 [64–153] days in the active mode; *P* = 0.06). Details of LLT regimen after the alert triggered in both groups are presented in Supplementary Table 2.

**Table 2:** Association of active mode CDS alert trigger with primary and secondary outcomes

Outcome and parameters	*N* (%)		Estimate (95% CI) or odds ratio (95% CI)^a^	*P* Value
	Silent mode (*n* = 800)	Active mode (*n* = 800)		
Primary outcomes, mean ± SD^b^				
LDL-C levels, mg/dL	144.0 ± 41.8	137.4 ± 44.8	−6.6 (−10.7 to −2.5)	0.002
Change in LDL-C, mg/dL	−71.5 ± 52.0	−76.6 ± 54.7	−6.6 (−10.8 to −2.6)	0.002
Secondary outcomes, observed data^c^				
Any statin use	430 (53.8%)	477 (59.6%)	1.32 (1.08 to 1.61)	0.007
High-intensity statin use	181 (22.6%)	260 (32.5%)	1.78 (1.41 to 2.23)	<0.001
LLT initiation or intensification	384 (48.0%)	433 (54.1%)	1.30 (1.06 to 1.58)	0.01
Secondary cause screening	93 (11.6%)	156 (19.5%)	1.83 (1.38 to 2.42)	<0.001

*CDS* clinical decision support, *CI* confidence interval, *LDL-C* low-density lipoprotein cholesterol, *SD* standard deviation.

^a^Parameter estimate (from linear regression models) or odds ratio (from logistic regression models) for active mode alert compared to silent mode alert.

^b^An imputation model was fit, where the dependent (Y) variable was change in LDL-C and the independent (X) variables were LDL-C levels at alert and change in LLT intensity in the 3 months after alert. Linear regression models for LDL-C levels in the 1–12 months after alert and for change in LDL-C (levels in the 1–12 months after alert minus levels at alert), adjusted for LDL-C level at alert, age at alert, male sex, coronary heart disease, and diabetes were then fit for all 1600 patients, using the observed LDL-C (or change in LDL-C) values where available, or the predicted values from the imputation models where missing. The process was repeated over 1000 bootstrap samples of 1600 randomly selected patients with replacement.

^c^Logistic regression models for each secondary outcome (statin use or LLT initiation or intensification in the 3 months after alert, or screening for secondary causes of hypercholesterolemia in the 30 days after alert) adjusted for age at alert, male sex, coronary heart disease, and diabetes.

The odds of screening for secondary causes of hypercholesterolemia were higher in active mode compared to silent mode (OR, 1.83; 95% CI, 1.38–2.42; *P* < 0.001). The frequency of clinicians ordering these laboratory tests in the 1-year prior to the alert trigger was similar in both groups (57.6% in active mode and 53.8% in silent mode, *P* = 0.12) likely reflecting routine patient testing. However, within 30 days of alert trigger, ordering was more frequent in the active mode group than in the silent mode group (19.5 vs. 11.6%; *P* < 0.001) (Supplement Table 3).

For alerts that triggered in the primary care setting (622 in silent mode, 674 in active mode), and those that triggered for physicians (598 in silent mode, 592 in active mode), subset analyses results were similar to the overall study results (Table 3). Among individuals who triggered the alert in a subspecialty clinic setting (178 in silent mode, 125 in active mode), there were no significant differences in the outcomes, however, the sample size was relatively small. For the subset of alerts that triggered for non-physician care providers (202 in silent mode, 207 in active mode), no significant associations were noted with the primary outcomes, while the results for the secondary outcomes were similar to the overall study results (Table 3). Alerts that triggered in urban (398 in silent mode, 382 in active mode) as well as rural centers (402 in silent mode, 417 in active mode), were associated with the primary outcome, more strongly in the latter setting where associations were additionally noted with secondary outcomes including initiation/intensification of LLT.

**Table 3:** Association of active mode CDS alert trigger with primary and secondary outcomes; stratified by department of practice, type of clinician, and location of medical facility

Outcome and parameters	*N* (%)		Estimate (95% CI) or odds ratio (95% CI)^b^	*P* Value
	Silent mode	Active mode^a^		
Primary care				
Primary outcomes, *n*^c^	622	674		
LDL-C levels, mean ± SD, mg/dL	141.3 ± 39.9	134.3 ± 44.2	−7.1 (−11.3 to −3.2)	<0.001
Change in LDL-C, mean ± SD, mg/dL	−68.6 ± 42.5	−75.2 ± 46.7	−7.1 (−11.0 to −3.2)	<0.001
Secondary outcomes, observed data, *n*^d^	622	674		
Any statin use	339 (54.5%)	416 (61.7%)	1.37 (1.10 to 1.72)	0.005
High-intensity statin use	137 (22.0%)	228 (33.8%)	1.91 (1.48 to 2.45)	<0.001
LLT initiation or intensification	307 (49.4%)	378 (56.1%)	1.32 (1.06 to 1.64)	0.01
Secondary cause screening	64 (10.3%)	138 (20.5%)	2.26 (1.64 to 3.11)	<0.001
Subspeciality clinic				
Primary outcomes, *n*^c^	178	125		
LDL-C levels, mean ± SD, mg/dL	155.2 ± 46.8	153.5 ± 45.0	−0.8 (−4.8 to 4.0)	0.80
Change in LDL-C, mean ± SD, mg/dL	−60.9 ± 47.3	−58.3 ± 46.7	−0.8 (−4.4 to 4.0)	0.83
Secondary outcomes, observed data, *n*^d^	178	125		
Any statin use	91 (51.1%)	61 (48.8%)	0.99 (0.62 to 1.58)	0.96
High-intensity statin use	44 (24.7%)	32 (25.6%)	1.17 (0.67 to 2.03)	0.58
LLT initiation or intensification	77 (43.3%)	54 (43.2%)	1.04 (0.65 to 1.67)	0.86
Secondary cause screening	29 (16.3%)	18 (14.4%)	0.84 (0.44 to 1.59)	0.59
MD/DO				
Primary outcomes, *n*^c^	598	592		
LDL-C levels, mean ± SD, mg/dL	143.5 ± 40.3	135.7 ± 44.8	−7.9 (−11.9 to −3.6)	0.002
Change in LDL-C, mean ± SD, mg/dL	−67.8 ± 45.1	−72.9 ± 46.6	−7.9 (−12.3 to −3.7)	0.002
Secondary outcomes, observed data, *n*^d^	598	592		
Any statin use	322 (53.8%)	351 (59.3%)	1.31 (1.04 to 1.65)	0.02
High-intensity statin use	142 (23.7%)	187 (31.6%)	1.62 (1.25 to 2.11)	<0.001
LLT initiation or intensification	293 (49.0%)	315 (53.2%)	1.22 (0.97 to 1.53)	0.10
Secondary cause screening	73 (12.2%)	108 (18.2%)	1.59 (1.15 to 2.20)	0.005
Non-MD/DO (NP or PA)				
Primary outcomes, *n*^c^	202	207		
LDL-C levels, mean ± SD, mg/dL	144.5 ± 45.4	141.8 ± 44.2	−3.4 (−7.4 to 0.7)	0.10
Change in LDL-C, mean ± SD, mg/dL	−66.7 ± 44.1	−71.4 ± 48.3	−3.4 (−8.0 to 0.7)	0.10
Secondary outcomes, observed data, *n*^d^	202	207		
Any statin use	108 (53.5%)	126 (60.9%)	1.34 (0.90 to 1.99)	0.16
High-intensity statin use	39 (19.3%)	73 (35.3%)	2.28 (1.43 to 3.62)	<0.001
LLT initiation or intensification	91 (45.0%)	117 (56.5%)	1.53 (1.03 to 2.27)	0.04
Secondary cause screening	20 (9.9%)	48 (23.2%)	2.63 (1.49 to 4.65)	<0.001
Academic medical center (urban)^e^				
Primary outcomes, *n*^c^	398	382		
LDL-C levels, mean ± SD, mg/dL	148.7 ± 42.9	142.7 ± 43.4	−5.7 (−9.7 to −1.6)	0.008
Change in LDL-C, mean ± SD, mg/dL	−64.7 ± 45.9	−66.8 ± 47.8	−5.7 (−9.7 to −1.6)	0.008
Secondary outcomes, observed data, *n*^d^	398	382		
Any statin use	207 (52.0%)	202 (52.9%)	1.09 (0.82 to 1.45)	0.56
High-intensity statin use	88 (22.1%)	115 (30.1%)	1.71 (1.22 to 2.39)	0.002
LLT initiation or intensification	182 (45.7%)	181 (47.4%)	1.07 (0.95 to 1.21)	0.51
Secondary cause screening	56 (14.1%)	69 (18.1%)	1.35 (0.91 to 1.98)	0.13
Health system clinic (rural)^f^				
Primary outcomes, *n*^c^	402	417		
LDL-C levels, mean ± SD, mg/dL	139.4 ± 40.2	132.5 ± 45.6	−7.6 (−11.7 to −3.6)	<0.001
Change in LDL-C, mean ± SD, mg/dL	−69.7 ± 42.0	−77.6 ± 45.8	−7.6 (−11.7 to −3.8)	<0.001
Secondary outcomes, observed data, *n*^d^	402	417		
Any statin use	223 (55.5%)	275 (65.9%)	1.60 (1.20 to 2.13)	0.001
High-intensity statin use	93 (23.1%)	145 (34.8%)	1.81 (1.32 to 2.47)	<0.001
LLT initiation or intensification	202 (50.2%)	251 (60.2%)	1.51 (1.14 to 1.99)	0.004
Secondary cause screening	37 (9.2%)	87 (20.9%)	2.63 (1.74 to 3.99)	<0.001

*CDS* clinical decision support, *CI* confidence interval, *DO* doctor of osteopathic medicine, *LDL-C* low-density lipoprotein cholesterol, *MD* doctor of medicine, *NP* nurse practitioner, *PA* physician assistant, *SD* standard deviation.

^a^One patient in the active mode group with unknown department of practice and unknown qualifications of provider was excluded from these analyses.

^b^Parameter estimate (from linear regression models) or odds ratio (from logistic regression models) for active mode alert compared to silent mode alert.

^c^An imputation model was fit, where the dependent (Y) variable was change in LDL-C and the independent (X) variables were LDL-C levels at alert and change in LLT intensity in the 3 months after alert. Linear regression models for LDL-C levels in the 1–12 months after alert and for change in LDL-C (levels in the 1–12 months after alert minus levels at alert), adjusted for LDL-C level at alert, age at alert, male sex, coronary heart disease, and diabetes were then fit for all patients in each subset, using the observed LDL-C (or change in LDL-C) values where available, or the predicted values from the imputation models where missing. The process was repeated over 1000 bootstrap samples of randomly selected patients with replacement.

^d^Logistic regression models for each secondary outcome (statin use or LLT initiation or intensification in the 3 months after alert, or screening for secondary causes of hypercholesterolemia in the 30 days after alert) adjusted for age at alert, male sex, coronary heart disease, and diabetes.

^e^Academic medical centers located in 3 urban areas: Rochester, Minnesota; Phoenix and Scottsdale, Arizona; and Jacksonville, Florida.

^f^Health system clinics located in several small towns/rural communities in Minnesota and Wisconsin.

## Discussion

We developed a CDS tool to facilitate management of severe hypercholesterolemia/possible FH in a health system comprising 3 main sites, 16 hospitals and 53 clinics. Using a pre-post implementation study design, we demonstrated that deployment of CDS was associated with clinician actions (initiation or intensification of LLT) and patient outcomes (lowering of LDL-C levels). Patient LDL-C levels within 1–12 months after the alert triggered were 6.6 mg/dL lower in active mode than silent mode (Table 2). The alert increased adherence to the ACC/AHA guideline with a nearly 50% relative increase (32.5 vs. 22.6%; *P* < 0.001) in the proportion of patients on a high-intensity statin within a 3-month period following alert trigger (Table 2).

The impact of the CDS alert was further highlighted by the resulting increase in ordering of laboratory tests (11.6 vs. 19.5%; *P* < 0.001) to rule out secondary causes of hypercholesterolemia (Table 2). In exploratory stratified analyses, CDS implementation was associated with lower LDL-C levels in the primary care setting, not in the specialty setting, and when the alert triggered for physicians, and not for non-physician care providers (Table 3). These results may be affected by attrition in the sample size in stratified analyses and larger study cohorts are needed to establish whether the effect of the CDS tool on outcomes is context and provider dependent.

The present study is, to our knowledge, the first to demonstrate that implementation of a CDS tool for severe hypercholesterolemia/possible FH was associated with lowering of LDL-C. Prior studies of CDS for hypercholesterolemia were limited to patients with CHD or poorly controlled diabetes^18–20^. In a systematic review of 41 studies of CDS and cardiovascular outcomes, the majority of studies (~75%) reported no improvement in outcomes^21^. A recent article summarized the key problems encountered in previous CDS studies and made recommendations for reporting of CDS studies^22^. The present study was aligned with many of these recommendations, in particular, incorporating clinician feedback in developing CDS.

Randomized controlled trials are expensive, time consuming, variably generalizable to the population of interest, and often there is a considerable lag time before results are applied in clinical practice^23^. The high impact of such trials, however, results from the ability to infer causality from the results. Although our study design was non-randomized, we did match the two comparator groups for known potential confounders, and our confidence in attribution of the outcome (lower LDL-C levels) to the intervention (CDS for elevated LDL-C) is heightened by the observation that there was a nearly 50% relative increase in the use of high-intensity statins in the active mode group following CDS implementation.

Preparatory to the present study, we used an implementation science framework and stakeholder-guidance to develop the CDS tool, increase usability and minimize alert fatigue and alert dismissal^14,15,24,25^. The resulting clinician actions and patient outcomes were captured during routine clinical care to generate evidence^24,26–29^. Our study is an example of a pragmatic intervention study in a large health system with disparate practice settings (geographic location, rural vs. urban, inpatient vs. outpatient) conducted within a relatively short time and in a relatively inexpensive manner. The study illustrates the potential of population health management using a learning health system^29,30^. As part of our pragmatic study design, we did not include a concurrent control group to avoid potential bias or carry-over effects from clinicians getting used to viewing the alert in the EHR. Clinicians could become either more aware to look for elevated LDL-C values or conversely, less aware, given they would rely on the alert to trigger in the setting of an elevated result. We included a sequential control group and data was collected contiguously for 3 months each, for the silent and active mode periods, minimizing the possibility that trends such as due to general changes in medical practice (e.g., changes in practice guidelines) or changes in institutional operations and/or policies could have led to the study findings.

Because of the non-randomized nature of our study, clinicians were not required to order follow-up lipid measurements for all patients. As a result, follow up LDL-C data was often not available, particularly in the silent mode group where the alert could not be viewed by clinicians in the EHR and LLT intensification was less likely. To overcome bias, LDL-C levels were imputed when missing. Since there was a nearly 50% relative increase in the use of high-intensity statins in the active mode group following CDS implementation, post-imputation LDL-C levels mitigate bias due to the non-random nature of the missing data and are more reflective of the true findings. The effect of CDS implementation on lowering LDL-C levels was relatively modest and a treatment gap remained, with 37.6% of patients in the active mode group and 43.0% of patients in the silent mode group not on LLT within 3 months after the alert triggered (Supplementary Table 2). Further work is needed to identify reasons for this remaining treatment gap and develop approaches to overcome it. A majority of the patients were white by self-report and there is a need to implement CDS in diverse settings and evaluate its impact on both patient and clinician outcomes. In both modes, 64% of the patients identified as female, likely due to the known influence of gender on health-seeking behavior with women more likely to seek medical care than men. Our study design was pragmatic in nature with sequential data collection which could have introduced variability due to seasonal influences; however we collected data for two contiguous 3-month periods minimizing the possibility that general trends led to the study findings.

Deployment of a CDS alert in a large health system, for severe hypercholesterolemia/possible FH (LDL-C ≥ 190 mg/dL) was associated with lowering of patient LDL-C levels, likely due to clinician led initiation/intensification of LLT. The alert was also associated with a nearly 50% relative increase in the proportion of patients on a high-intensity statin within a 3-month period following alert trigger. Our results suggest that deploying CDS developed using an implementation science framework and incorporating clinician feedback has the potential to optimize patient management related to cardiovascular risk factors.

## Methods

This study was conducted as a quality improvement project at Mayo Clinic from August 2018 to January 2023 and was therefore considered exempt by the Mayo Clinic Institutional Review Board. We excluded patients who had declined authorization for review of their medical records for research purposes. We followed the Transparent Reporting of Evaluations with Nonrandomized Designs (TREND) guidelines for nonrandomized evaluations of public health interventions (Supplementary Table 4)^31,32^.

### Development of the CDS tool for possible FH

We developed an electronic phenotyping algorithm to identify individuals with severe hypercholesterolemia/possible FH (Table 4) and linked it to a CDS tool in the EHR, as previously described^12–15^. The CDS triggered when a patient met the following criteria: LDL-C ≥ 190 mg/dL, age 18–80 years, and no identified secondary causes of hypercholesterolemia (such as hypothyroidism, cholestasis, or nephrotic syndrome) or mixed hyperlipidemia (triglyceride ≥ 400 mg/dL). Additionally, the CDS alert was configured so as not to trigger for patients with a known FH diagnosis or existing genetic testing results for FH in the EHR.

**Table 4:** EHR-based algorithm to detect severe hypercholesterolemia/possible FH

FH algorithm criteria	Result value	Data element	Code(s)
LDL-C	≥ 190 mg/dL	LOINC codes	2089-1, 2090-9, 12773-8, 13457-7, 18261-8, 18262-6, 22748-8, 35198-1, 39469-2, 49132-4, 55440-2, 69419-0
Age at alert trigger	18–80 years	Demographic data	
No disqualifying values for secondary causes of hypercholesterolemia	TSH < 10 mIU/L	LOINC codes	3016-3, 11579-0, 14999-7
	ALP < 200 IU/L	LOINC codes	6768-6
	Urine protein < 3000 mg/24 h	LOINC codes	2889-4
No documented FH diagnosis	Hypercholesterolemia Familial Hyperlipidemia IIa	ICD10-CM code	E78.01
No prior or active Mayo FH Clinic order	Cardiovascular Disease Lipid (Cardiology) Consult (Clinic)	Administrative data	
No disqualifying values for triglycerides	< 400 mg/dL	LOINC codes	1644-4, 2571-8, 3043-7, 3048-6, 12951-0
No prior or active Mayo FH gene panel order	Familial hypercholesterolemia and related disorders multi-gene panel	CPT codes	81406, 81407, 81479

*ALP* alkaline phosphatase, *CPT* Current Procedural Terminology, *FH* familial hypercholesterolemia, *ICD10-CM* International Classification of Diseases, tenth revision, Clinical Modification, *LDL-C* low-density lipoprotein cholesterol, *LOINC* Logical Observation Identifier Names and Codes, *TSH* thyroid stimulating hormone.

The CDS was developed and iteratively refined using an implementation science framework and key stakeholder feedback from focus groups, semi-structured qualitative interviews, and surveys^13–15^. At each stage of CDS development, stakeholder feedback was sought to iteratively refine the alert, its linked phenotyping algorithm as well as its implementation in the EHR. Hasnie et al. conducted a survey of 210 clinicians and focus groups with 19 physicians to gather input on the structure and form of the initial CDS prototype^13^. Next, Bangash et al. conducted qualitative interviews and usability testing with 13 clinicians in primary care and cardiology to further refine the CDS content and interface design^15^. After the CDS was deployed in the EHR, a post-implementation survey of 104 clinicians across Mayo Clinic and the Health System was conducted to further refine implementation of the alert. Since clinicians were the primary targets of the CDS, we included them throughout the process of design and implementation to increase adoption of the tool in practice and ensure its harmonization with clinical workflows.

The CDS was designed as a point of care knowledge resource for clinicians and formatted as an asynchronous in-basket message linked to lipid panel results^14,15^. The in-basket message included recommendations for high-intensity statin initiation (as per the ACC/AHA guideline)^33^, ruling out secondary causes of hypercholesterolemia, consideration of genetic testing (particularly in the presence of concomitant personal history and/or family history of CHD)^34–37^, conducting cascade testing of at-risk family members, and the option to refer to a lipid specialist.

### Setting

The study was conducted at Mayo Clinic, an academic medical center with 3 main sites in Rochester, Minnesota, Jacksonville, Florida and Phoenix/Scottsdale, Arizona, as well as in the Mayo Clinic Health System, that serves primarily rural communities in Minnesota and Wisconsin through 16 hospitals and 53 clinics.

### Study design and patient population

We employed a pre-post implementation study design to investigate whether implementation of the CDS for severe hypercholesterolemia/possible FH was associated with lower LDL-C in patients with levels ≥ 190 mg/dL. The CDS was deployed in silent mode for three months prior to implementation, running in the background of the EHR from August 13 to November 12, 2020. Clinicians were unable to view the CDS during the silent mode period. The CDS then transitioned to active mode, defined as the post-implementation period, at which point clinicians were able to view the CDS in the EHR and engage with it when it triggered. The CDS ran in active mode for 3 months between November 13, 2020, to February 12, 2021 (Fig. 1).

We excluded individuals who had declined research participation (64 were excluded from the silent mode group and 57 from the active mode group) and 1 individual who had an erroneous LDL-C result, which was corrected to < 190 mg/dL at alert trigger. There were 24 individuals in whom the CDS alert triggered both in silent and active mode periods - they were included in the silent mode period only due to difficulty in the attribution of measured outcomes. Similarly, if an individual had multiple CDS alert triggers within a period, the earliest alert date was used for analyses. We then matched individuals in silent mode and active mode in a 1:1 ratio based on age (±5 years), sex, and baseline LLT regimen which was defined as any statin use, statin intensity, and number of LLT medication classes.

### Data collection

The silent mode and active mode periods lasted 3 months each. We ascertained demographic and clinical data at the alert trigger including age, sex, baseline LDL-C, tobacco use, body mass index, and history of diabetes, hypertension, and CHD, from the EHR (Supplementary Table 5). All LDL-C results starting at > 30 days after the alert but within 1 year of the alert trigger were obtained from the EHR. Orders for laboratory testing to screen for secondary causes of hypercholesterolemia within 1 year before the alert and within 30 days after the alert trigger were obtained from the EHR for those individuals who had not been screened for secondary causes in the prior year. LLT data including dates, medication class, intensity, and dosage were ascertained from the EHR for the period starting 30 days before until 3 months after the alert (Supplementary Table 6). Statin intensity was defined as low, medium, or high based on the ACC/AHA guideline for LLT^33^.

In exploratory analyses, we ascertained type of clinician (MD/DO vs. non-MD/DO) and department of practice (primary care or subspecialty care) in both modes to assess the effect of these variables on study outcomes.

### Outcomes assessed

The primary outcome was the difference in LDL-C levels between the silent and active mode groups within 1–12 months after the alert triggered. If patients had > 1 LDL-C measurement during this period, the earliest LDL-C value in the 1–12-month period was selected for analysis. As a sensitivity analysis, the latest LDL-C was also analyzed. LDL-C measurements < 1 month after the alert would be less likely attributable to the CDS alert as LLT can take ~2–4 weeks to reduce LDL-C levels. Therefore, we assessed the primary outcome starting 1 month after the alert trigger.

Secondary outcomes included any LLT initiation or intensification within 3 months after the alert trigger and clinician ordering of relevant laboratory tests to screen for secondary causes of hypercholesterolemia within 30 days after the alert trigger. Initiation of LLT was defined as new LLT for those individuals who were not on any prior LLT at the time of alert trigger and intensification was defined as an increase in statin dose/statin intensity or the addition of a new medication class to an existing LLT regimen. The laboratory tests included in the screening of secondary causes were serum alkaline phosphatase, thyroid function tests (thyroid stimulating hormone, triiodothyronine, and/or thyroxine) and urinary protein or urine protein/creatinine ratio to evaluate for liver disease, hypothyroidism, and nephrotic syndrome, respectively^12^.

### Sample size calculation

We chose 3-month periods before and after switching the alert to active mode, based on the observation that on average ~300 patients with possible FH were detected per month by the EHR algorithm. We assumed ~900 patients would be detected in each 3-month period and after exclusions we would have ~800 eligible patients. We assumed 25% would not have LDL-C levels available in the 1–12 month period after alert trigger. With a sample size of 600 in each group, we would be able to detect a difference in LDL-C levels of 6 mg/dL between the 2 groups, with at least 80% power, assuming an LDL-C standard deviation of 35 mg/dL.

### Statistical analyses

Comparisons between baseline variables in silent and active mode periods were performed using χ2 or Fisher’s exact tests for categorical variables and using *t*-tests or Wilcoxon rank sum tests for continuous variables. Multivariable linear regression models were used to assess the association between CDS implementation and each primary outcome, adjusted for age, sex, baseline LDL-C at the alert, CHD, and diabetes. Multivariable logistic regression models were used to assess the association between CDS implementation and each secondary outcome, adjusted for age, sex, CHD, and diabetes.

Missing LDL-C values in the follow-up period were imputed to avoid bias due to the nonrandom nature of missingness, using a prediction model that utilized pooled data (without regard to the group – silent mode or active mode) and included statin intensification. Specifically, a linear regression model was fit to predict follow-up LDL-C values adjusted for baseline LDL-C values and change in LLT intensity in the 3 months after alert. Then a multivariable linear regression model was fit to assess the association between CDS implementation and the (observed or imputed) LDL-C value (or change in LDL-C) in the 1–12 months after alert, adjusted for baseline LDL-C, age, sex, CHD, and diabetes. The imputation models and the primary outcome models were repeated 1000 times in randomly selected bootstrap samples to obtain confidence intervals and *P* values.

In exploratory analyses, we stratified encounters in both silent mode and active mode by clinician type (physicians and non-physician care providers) and department where the alert triggered (primary care and subspecialty care). We conducted analyses for the primary and secondary outcomes within each stratum separately, without regard to whether both members of matched pairs were in the strata.

All tests were two-sided, and *P* values < 0.05 were considered statistically significant. Analyses were performed using SAS software, version 9.4 (SAS Institute, Inc.).

### Reporting summary

Further information on research design is available in the Nature Research Reporting Summary linked to this article.

### Supplementary information

Supplement

Reporting Summary

## Data Availability

The data used and analyzed during the current study are available from the corresponding author on reasonable request.

## References

[1] Saadatagah S (2022). The burden of severe hypercholesterolemia and familial hypercholesterolemia in a population-based setting in the US. Am. J. Prev. Cardiol..

[2] Sniderman AD, Tsimikas S, Fazio S (2014). The severe hypercholesterolemia phenotype: clinical diagnosis, management, and emerging therapies. J. Am. Coll. Cardiol..

[3] Bucholz EM, Rodday AM, Kolor K, Khoury MJ, de Ferranti SD (2018). Prevalence and predictors of cholesterol screening, awareness, and statin treatment among US adults with familial hypercholesterolemia or other forms of severe dyslipidemia (1999-2014). Circulation.

[4] Gidding SS (2015). The agenda for familial hypercholesterolemia: A scientific statement from the American Heart Association. Circulation.

[5] de Ferranti SD (2016). Prevalence of familial hypercholesterolemia in the 1999 to 2012 United States National Health and Nutrition Examination Surveys (NHANES). Circulation.

[6] Versmissen J (2008). Efficacy of statins in familial hypercholesterolaemia: a long term cohort study. BMJ.

[7] Knowles JW (2014). Reducing the burden of disease and death from familial hypercholesterolemia: a call to action. Am. Heart J..

[8] Reiner Z (2015). Management of patients with familial hypercholesterolaemia. Nat. Rev. Cardiol..

[9] Ogura M (2018). PCSK9 inhibition in the management of familial hypercholesterolemia. J. Cardiol..

[10] Krähenbühl S, Pavik-Mezzour I, von Eckardstein A (2016). Unmet needs in LDL-C lowering: When statins won’t do!. Drugs.

[11] deGoma EM (2016). Treatment gaps in adults with heterozygous familial hypercholesterolemia in the United States: Data from the CASCADE-FH Registry. Circ. Cardiovasc. Genet..

[12] Safarova MS, Liu H, Kullo IJ (2016). Rapid identification of familial hypercholesterolemia from electronic health records: The SEARCH study. J Clin Lipidol.

[13] Hasnie AA, Kumbamu A, Safarova MS, Caraballo PJ, Kullo IJ (2018). A clinical decision support tool for familial hypercholesterolemia based on physician input. Mayo Clin. Proc. Innov. Qual. Outcomes.

[14] Bangash H (2020). Deploying clinical decision support for familial hypercholesterolemia. ACI Open.

[15] Bangash H (2020). An implementation science framework to develop a clinical decision support tool for familial hypercholesterolemia. J. Pers. Med..

[16] Farwati M, Kumbamu A, Kochan DC, Kullo IJ (2018). Patient and provider perspectives on a decision aid for familial hypercholesterolemia. J. Pers. Med..

[17] Bangash H (2022). Web-based tool (FH Family Share) to increase uptake of cascade testing for familial hypercholesterolemia: development and evaluation. JMIR Hum. Factors.

[18] Gilutz H (2009). Computerized community cholesterol control (4C): meeting the challenge of secondary prevention. Isr Med. Assoc. J..

[19] Ali MK (2016). Effectiveness of a multicomponent quality improvement strategy to improve achievement of diabetes care goals: A randomized, controlled trial. Ann. Intern. Med..

[20] Shi X (2023). Comparative effectiveness of team-based care with and without a clinical decision support system for diabetes management : A cluster randomized trial. Ann. Intern. Med..

[21] Lu Y, Melnick ER, Krumholz HM (2022). Clinical decision support in cardiovascular medicine. BMJ.

[22] Kawamoto K, McDonald CJ (2020). Designing, conducting, and reporting clinical decision support studies: Recommendations and call to action. Ann. Intern. Med..

[23] Angus DC (2015). Fusing randomized trials with big data: The key to self-learning health care systems?. JAMA.

[24] Bangash H (2023). Clinician perspectives on clinical decision support for familial hypercholesterolemia. J. Pers. Med..

[25] Ancker JS (2017). Effects of workload, work complexity, and repeated alerts on alert fatigue in a clinical decision support system. BMC Med. Inform. Decis. Mak..

[26] Maddox TM (2017). The learning healthcare system and cardiovascular care: A scientific statement from the American Heart Association. Circulation.

[27] Kullo IJ, Jarvik GP, Manolio TA, Williams MS, Roden DM (2013). Leveraging the electronic health record to implement genomic medicine. Genet. Med..

[28] Chambers DA, Feero WG, Khoury MJ (2016). Convergence of implementation science, precision medicine, and the learning health care system: A new model for biomedical research. JAMA.

[29] Lu CY (2018). A proposed approach to accelerate evidence generation for genomic-based technologies in the context of a learning health system. Genet. Med..

[30] Williams MS (2018). Patient-centered precision health in a learning health care system: Geisinger’s genomic medicine experience. Health Aff..

[31] Des Jarlais DC, Lyles C, Crepaz N (2004). Improving the reporting quality of nonrandomized evaluations of behavioral and public health interventions: the TREND statement. Am. J. Public Health.

[32] Simera I, Moher D, Hoey J, Schulz KF, Altman DG (2010). A catalogue of reporting guidelines for health research. Eur. J. Clin. Invest..

[33] Grundy SM (2019). 2018 AHA/ACC/AACVPR/AAPA/ABC/ACPM/ADA/AGS/APhA/ASPC/NLA/PCNA Guideline on the management of blood cholesterol: Executive summary: A report of the American College of Cardiology/American Heart Association Task Force on Clinical Practice Guidelines. J. Am. Coll. Cardiol..

[34] Nordestgaard BG (2013). Familial hypercholesterolaemia is underdiagnosed and undertreated in the general population: guidance for clinicians to prevent coronary heart disease: consensus statement of the European Atherosclerosis Society. Eur. Heart J..

[35] O’Brien EC (2014). Rationale and design of the familial hypercholesterolemia foundation CAscade SCreening for Awareness and DEtection of Familial Hypercholesterolemia registry. Am. Heart J..

[36] Lee C, Rivera-Valerio M, Bangash H, Prokop L, Kullo IJ (2019). New case detection by cascade testing in familial hypercholesterolemia: A systematic review of the literature. Circ. Genom. Precis. Med..

[37] Safarova MS, Kullo IJ (2016). My approach to the patient with familial hypercholesterolemia. Mayo Clin. Proc..

