# Supplementary material for: Effect of clinical decision support for severe hypercholesterolemia on low-density lipoprotein cholesterol levels
Source: NPJ Digit Med. 2024 Mar 18;7:73. doi: 10.1038/s41746-024-01069-w (PMC10948900; doi:10.1038/s41746-024-01069-w)
Supplement: Supplementary file 1 — Supplement [file 41746_2024_1069_MOESM1_ESM.pdf]

Effect of Clinical Decision Support for Severe Hypercholesterolemia on Low-Density Lipoprotein Cholesterol Levels

Supplemental Online Content

Bangash H, Saadatagah S, Naderian M, Hamed M, Alhalabi L, Sherafati A, Sutton J, Elsekaily O, Mir A, Gundelach JH, Gibbons D, Johnsen P, Wood-Wentz CM, Smith CY, Caraballo P, Bailey KR, Kullo IJ.

Table of Contents 2

This supplemental material has been provided by the authors to give readers additional information about their work.

Online Supplement Table of Contents

Supplement Table 1. Location of Medical Facility at CDS Alert Trigger 3
Supplement Table 2. LDL-C Testing and Lipid Lowering Therapy Regimen After the CDS Alert Triggered 4
Supplement Table 3. Evaluation of Secondary Causes of Hypercholesterolemia 5
Supplement Table 4. Completed TREND Checklist 6
Supplement Table 5. Diagnostic and Procedural Codes Used to Ascertain Patient Comorbidities 10
Supplement Table 6. RxCUI Codes to Ascertain Lipid Lowering Therapy 11

Supplement Table 1. Location of Medical Facility at CDS Alert Trigger

Type of Facility	State	City(s)	N (%) Silent Mode (n = 800)	N (%) Active Mode (n = 800)
Academic medical center				
Primary care	Minnesota	Rochester	124 (15.5%)	127 (15.9%)
Subspecialty clinic			70 (8.8%)	48 (27.4%)
Academic medical center				
Primary care	Arizona	Phoenix, Scottsdale	70 (8.8%)	82 (10.3%)
Subspecialty clinic			36 (4.5%)	25 (3.1%)
Academic medical center				
Primary care	Florida	Jacksonville	88 (11.0%)	102 (12.8%)
Subspecialty clinic			38 (4.8%)	32 (4.0%)
Health system	Minnesota	Adams, Albert Lea, Austin, Belle Plaine, Cannon Falls, Ellsworth, Fairmont, Faribault, Janesville, Lake City, Lake Mills, Le Sueur, Mankato, Montgomery, New Prague, New Richland, Owatonna, Plainview, Red Wing, St. James, St. Peter, Waseca, Waterville, Wells, Zumbrota		
Primary care			176 (22.0%)	166 (20.8%)
Subspecialty clinic			18 (2.3%)	11 (1.4%)
Health system	Wisconsin	Arcadia, Barron, Bloomer, Caledonia, Chetek, Chippewa Falls, Clairemont, Eau Claire, Elmwood, Glenwood, Holmen, La Crosse, Menomonie, Mondovi, Onalaska, Osseo, Prairie du Chien, Rice Lake, Sparta, Tomah		
Primary care			164 (20.5%)	197 (24.6%)
Subspecialty clinic			16 (2.0%)	9 (1.1%)
Unknown			0 (0.0%)	1 (0.1%)

Supplement Table 2. LDL-C Testing and Lipid Lowering Therapy Regimen After the CDS Alert Triggered

Characteristic	N (%)		P Value[a]
	Silent Mode (n = 800)	Active Mode (n = 800)	
LDL-C testing in 1-12 months after the alert			
Any testing	415 (51.9%)	484 (60.5%)	<0.001
Number of tests, median (range)	1 (1, 7)	1 (1, 11)	0.78
LDL-C, mean (SD), mg/dL[b]	139.7 (51.4)	133.9 (52.3)	0.10
Change in LDL-C, mean (SD), mg/dL[c]	-71.5 (52.0)	-76.6 (54.7)	0.15
LLT regimen in 3 months after the alert			
Number of medication classes[d]			0.04
0	344 (43.0%)	301 (37.6%)	
1	422 (52.8%)	475 (59.4%)	
2	31 (3.9%)	21 (2.6%)	
3	3 (0.4%)	3 (0.4%)	
Statin use	430 (53.8%)	477 (59.6%)	0.02
Statin intensity			<0.001
No statin use	370 (46.3%)	323 (40.4%)	
Low	22 (2.8%)	12 (1.5%)	
Medium	227 (28.4%)	205 (25.6%)	
High	181 (22.6%)	260 (32.5%)	
Change in statin intensity[e]			0.008
No change	322 (40.3%)	284 (35.5%)	
1 level	35 (4.4%)	33 (4.1%)	
2 levels	236 (29.5%)	213 (26.6%)	
3 levels	207 (25.9%)	270 (33.8%)	
Non statin use	58 (7.3%)	45 (5.6%)	0.19
LLT initiation or intensification	392 (49.0%)	444 (55.5%)	0.009
Type			0.02
None	408 (51.0%)	356 (44.5%)	
Initiation	372 (46.5%)	411 (51.4%)	
Increase statin intensity or dose	19 (2.4%)	31 (3.9%)	
Addition of medication class	1 (0.1%)	2 (0.3%)	

[a]*P* values were calculated using chi-square tests, Fisher's exact tests, Wilcoxon rank sum tests, or *t*-tests.

[b]If a patient had more than one LDL-C test in the 1-12 months after the alert, the earliest result was reported.

[c]Calculated as the earliest LDL-C measurement in the 1-12 months after the alert minus the LDL-C measurement at the alert trigger.

[d]Medication classes include statins, bile acid sequestrants, ezetimibe, fibrates, monoclonal antibodies, niacin, and other non-statins.

[e]Comparison of statin intensity in the 3 months after the alert to the statin intensity at the alert. For example, high-intensity statin use in the 3 months after the alert and no statin use at the alert trigger would be classified as a change of 3 levels.

CDS = clinical decision support; LDL-C = low-density lipoprotein cholesterol; LLT = lipid lowering therapy; SD = standard deviation.

Supplement Table 3. Evaluation of Secondary Causes of Hypercholesterolemia

Secondary Cause	N (%)		*P* Value[a]
	Silent Mode (n = 800)	Active Mode (n = 800)	
Testing in 12 months before the alert			
Alkaline phosphatase	60 (7.5%)	72 (9.0%)	0.28
Thyroid function (thyroid stimulating hormone, triiodothyronine, and/or thyroxine)	408 (51.0%)	440 (55.0%)	0.11
Urinary protein or urine protein/creatinine ratio	46 (5.8%)	36 (4.5%)	0.26
Any of the above	430 (53.8%)	461 (57.6%)	0.12
Testing in 30 days after the alert			
Alkaline phosphatase	16 (2.0%)	55 (6.9%)	<0.001
Thyroid function (thyroid stimulating hormone, triiodothyronine, and/or thyroxine)	67 (8.4%)	128 (16.0%)	<0.001
Urinary protein or urine protein/creatinine ratio	18 (2.3%)	17 (2.1%)	0.86
Any of the above	93 (11.6%)	156 (19.5%)	<0.001

[a]*P* values were calculated using chi-square tests.

Supplement Table 4. Completed TREND Checklist

	Item No	Descriptor	Reported? ✓	Pg #
Title and Abstract				
Title and Abstract	1	Information on how unit were allocated to interventions	✓	1
		Structured abstract recommended	✓	1
		Information on target population or study sample	✓	1
Introduction				
Background	2	Scientific background and explanation of rationale	✓	1-2
		Theories used in designing behavioral interventions	✓	1-2
Methods				
Participants	3	Eligibility criteria for participants, including criteria at different levels in recruitment/sampling plan (e.g., cities, clinics, subjects)	✓	6-7
		Method of recruitment (e.g., referral, self-selection), including the sampling method if a systematic sampling plan was implemented	✓	6-7
		Recruitment setting	✓	6-7
		Settings and locations where the data were collected	✓	6-7 and Suppl Table 1
Interventions	4	Details of the interventions intended for each study condition and how and when they were actually administered, specifically including:	✓	6-7
		o Content: what was given?	✓	6-7
		o Delivery method: how was the content given?	✓	7
		o Unit of delivery: how were the subjects grouped during delivery?	✓	7
		o Deliverer: who delivered the intervention?	✓	7
		o Setting: where was the intervention delivered?	✓	7 and Suppl Table 1
		o Exposure quantity and duration: how many sessions or episodes or events were intended to be delivered? How long were they intended to last?	✓	7
		o Time span: how long was it intended to take to deliver the intervention to each unit?	✓	7
		o Activities to increase compliance or adherence (e.g., incentives)	N/A	-
Objectives	5	Specific objectives and hypotheses	✓	7-8
Outcomes	6	Clearly defined primary and secondary outcome measures	✓	7-8
		Methods used to collect data and any methods used to enhance the quality of measurements	✓	8
		Information on validated instruments such as psychometric and biometric properties	✓	8

Sample Size	7	How sample size was determined and, when applicable, explanation of any interim analyses and stopping rules	✓	8
Assignment Method	8	Unit of assignment (the unit being assigned to study condition, e.g., individual, group, community)	✓	7-8
		Method used to assign units to study conditions, including details of any restriction (e.g., blocking, stratification, minimization)	✓	7-8
		Inclusion of aspects employed to help minimize potential bias induced due to non-randomization (e.g., matching)	✓	7-8
Blinding (masking)	9	Whether or not participants, those administering the interventions, and those assessing the outcomes were blinded to study condition assignment; if so, statement regarding how the blinding was accomplished and how it was assessed.	N/A	-
Unit of Analysis	10	Description of the smallest unit that is being analyzed to assess intervention effects (e.g., individual, group, or community)	✓	7-8
		If the unit of analysis differs from the unit of assignment, the analytical method used to account for this (e.g., adjusting the standard error estimates by the design effect or using multilevel analysis)	N/A	-
Statistical Methods	11	Statistical methods used to compare study groups for primary methods outcome(s), including complex methods of correlated data	✓	8
		Statistical methods used for additional analyses, such as a subgroup analyses and adjusted analysis	✓	8
		Methods for imputing missing data, if used	✓	8
		Statistical software or programs used	✓	8
Results				
Participant flow	12	Flow of participants through each stage of the study: enrollment, assignment, allocation, and intervention exposure, follow-up, analysis (a diagram is strongly recommended)	✓	2 and Figure 1
		o Enrollment: the numbers of participants screened for eligibility, found to be eligible or not eligible, declined to be enrolled, and enrolled in the study	✓	2 and Figure 1
		o Assignment: the numbers of participants assigned to a study condition	✓	2 and Figure 1
		o Allocation and intervention exposure: the number of participants assigned to each study condition and the number of participants who received each intervention	✓	2 and Figure 1
		o Follow-up: the number of participants who completed the follow- up or did not complete the follow-up (i.e., lost to follow-up), by study condition	✓	2 and Figure 1
		o Analysis: the number of participants included in or excluded from the main analysis, by study condition	✓	2 and Figure 1
		Description of protocol deviations from study as planned, along with reasons	N/A	-
Recruitment	13	Dates defining the periods of recruitment and follow-up	✓	7

Baseline Data	14	Baseline demographic and clinical characteristics of participants in each study condition	✓	2 and Table 1
		Baseline characteristics for each study condition relevant to specific disease prevention research	✓	2 and Table 1
		Baseline comparisons of those lost to follow-up and those retained, overall and by study condition	✓	2 and Table 1
		Comparison between study population at baseline and target population of interest	✓	2 and Table 1
Baseline equivalence	15	Data on study group equivalence at baseline and statistical methods used to control for baseline differences	✓	2 and Table 1
Numbers analyzed	16	Number of participants (denominator) included in each analysis for each study condition, particularly when the denominators change for different outcomes; statement of the results in absolute numbers when feasible	✓	2 and Figure 1
		Indication of whether the analysis strategy was "intention to treat" or, if not, description of how non-compliers were treated in the analyses	N/A	-
Outcomes and estimation	17	For each primary and secondary outcome, a summary of results for each estimation study condition, and the estimated effect size and a confidence interval to indicate the precision	✓	2-3
		Inclusion of null and negative findings	✓	2-3
		Inclusion of results from testing pre-specified causal pathways through which the intervention was intended to operate, if any	N/A	-
Ancillary analyses	18	Summary of other analyses performed, including subgroup or restricted analyses, indicating which are pre-specified or exploratory	✓	3 and Table 3
Adverse events	19	Summary of all important adverse events or unintended effects in each study condition (including summary measures, effect size estimates, and confidence intervals)	N/A	-
Discussion				
Interpretation	20	Interpretation of the results, taking into account study hypotheses, sources of potential bias, imprecision of measures, multiplicative analyses, and other limitations or weaknesses of the study	✓	3-6
		Discussion of results taking into account the mechanism by which the intervention was intended to work (causal pathways) or alternative mechanisms or explanations	✓	3-6
		Discussion of the success of and barriers to implementing the intervention, fidelity of implementation	✓	3-6
		Discussion of research, programmatic, or policy implications	✓	3-6
Generalizability	21	Generalizability (external validity) of the trial findings, taking into account the study population, the characteristics of the intervention, length of follow-up, incentives, compliance rates, specific sites/settings involved in the study, and other contextual issues	✓	3-6
Overall Evidence	22	General interpretation of the results in the context of current evidence and current theory	✓	3-6

From: Des Jarlais DC, Lyles C, Crepaz N, the Trend Group. Improving the reporting quality of nonrandomized evaluations of behavioral and public health interventions: The TREND statement. *Am J Public Health*. 2004;94: 361-366. For more information, visit: http://www.cdc.gov/trendstatement/

Supplement Table 5. Diagnostic and Procedural Codes Used to Ascertain Patient Comorbidities

Comorbidity	Code Type	Codes
Diabetes mellitus[a]	ICD-9 (diagnostic)	250.xx
	ICD-10 (diagnostic)	E10.xxxx, E11.xxxx
Hypertension[a]	ICD-9 (diagnostic)	401.x, 402.xx, 403.xx, 404.xx, 405.xx
	ICD-10 (diagnostic)	I10, I11.x, I12.x, I13.xx, I15.x, I16.x
Coronary heart disease[b]	ICD-9 (diagnostic)	410.xx, 412, 429.7x, V45.81, V45.82
	ICD-10 (diagnostic)	I21.01, I21.02, I21.09, I21.11, I21.19, I21.21, I21.29, I21.3, I21.4, I22.x, I23.x8, I24.1, I25.2, Z95.1, Z95.5, Z98.61
	ICD-9 (procedural)	00.66, 36.0x, 36.1x, 36.2
	ICD-10 (procedural)	0210xxx, 0211xxx, 0212xxx, 0213xxx, 0270xxx, 0271xxx, 0272xxx, 0273xxx, 02C0xxx, 02C1xxx, 02C2xxx, 02C3xxx, 3E07017, 3E070PZ, 3E07317, 3E073PZ
	CPT-4 (procedural)	33510, 33511, 33512, 33513, 33514, 33516, 33517, 33518, 33519, 33521, 33522, 33523, 33533, 33534, 33535, 33536, 92920, 92921, 92924, 92925, 92928, 92929, 92933, 92934, 92937, 92938, 92941, 92943, 92944, 92973, 92974, 92980, 92981, 92982, 92984, 92995, 92996, 92973, 92974

[a]Defined as 2 or more instances of diagnostic codes.

[b]Defined as 3 or more instances of diagnostic codes or 1 or more instances of procedural codes.

CPT-4 = Current Procedural Terminology 4th edition; ICD-9 = International Classification of Diseases, Ninth revision; ICD-10 = International Classification of Diseases, Tenth revision.

Supplement Table 6. RxCUI Codes to Ascertain Lipid Lowering Therapy

Ingredient Name(s)	Medication Class(es)	RxCUI Codes
Alirocumab	Monoclonal antibody	1659152-1659161, 1659164-1659167, 1659176-1659179, 1659182, 1659183
Atorvastatin	Statin	83366-83367, 153165, 259255, 262095, 329299, 367665, 370621, 574676, 597966, 597970, 597983, 617310-617314, 617317-617320, 1158284-1158285, 1177400-1177401, 1297766, 1483793, 2630757-2630758, 2630760, 2631862-2631865, 2631867
Atorvastatin / amlodipine besylate	Statin	404011, 404013, 404773, 404914, 405788, 597967, 597971, 597974, 597977, 597980, 597984, 597987, 597990, 597993, 750197, 750199, 750201, 750203, 750205, 750207, 750209, 750211, 750213, 750215, 750217-750219, 750221, 750223, 750225, 750227, 750229, 750231, 750233, 750235, 750237, 750239, 1152281-1152282, 1170193, 1170826
Atorvastatin / ezetimibe	Statin, Ezetimibe	1422082-1422086, 1422093, 1422096, 1422099, 2611243-2611254
Bempedoic acid	Other non-Statin	2282403-2282414
Bempedoic acid / ezetimibe	Other non-Statin, Ezetimibe	2283226-2283236
Cholestyramine	Bile acid sequestrant	2447, 202582, 219397, 317329, 848943, 848949, 848951, 1156140, 1177645, 1185876, 1801265-1801266, 1801268-1801270, 1801272-1801274, 1801279-1081280
Colesevelam	Bile acid sequestrant	141625-141626, 284918, 368175, 374632, 866900, 866903, 866905, 866907, 866909-866912, 1164984-1164985, 1187941-1187942, 1870336-1870338, 1870340-1870342, 1870344, 1870346, 2123382-2123387
Colestipol	Bile acid sequestrant	2685, 104485, 151533, 368090, 374415, 1048444-1048447, 1048449-1048452, 1155486-1155487, 1166289-1166290, 1295303, 1296445, 2100170, 2100172
Evolocumab	Monoclonal antibody	1665684, 1665892-1665900, 1665903-1665906, 1801317-1801322
Ezetimibe	Ezetimibe	341248, 349556, 350671, 352304, 353099, 368566, 378303, 576000, 1163793-1163794, 1187441-1187442
Fenofibrate	Fibrate	8703, 141916, 200311, 220456, 221100, 310288-310289, 334738, 349287, 351133, 368613, 379814-379816, 379818, 379821-379823, 389191, 393452, 439656, 477560-477563, 483424-483429, 540280-540281, 544517-544518, 577030-577031, 578784, 578795-578799, 583093-583096, 615906, 616851-616853, 702052, 702054-702055, 702168-702169, 749801-749804, 763247-763252, 817139, 1159042-1159043, 1165999, 1166487-1166488, 1176848, 1177402-1177403, 1182714-1182715, 1183240-1183241, 1442162-1442165, 1442167-1442170
Fenofibric acid	Fibrate	24852, 828372-828381, 860879-860888, 1168314-1168315, 1183781-1183782
Fluvastatin	Statin	41127, 72875, 103918-103919, 151972, 310404-310405, 328265, 328267, 330788, 360507, 366775, 372255, 375074, 563452-563453, 687046-687048, 1165660-1165661, 1166061-1166062
Gemfibrozil	Fibrate	4719, 202999, 205751, 310459, 315106, 315976, 317636, 369510, 372299-372300, 566639, 1163944-1163945, 1179662-1179663
Lomitapide	Other non-Statin	1364478-1364498, 1648769-1648780
Lovastatin	Statin	6472, 197903-197905, 206257-206258, 224938, 316174-316176, 353377, 359731-359732, 368931, 372652, 378724, 433849, 541841, 541843,

Ingredient Name(s)	Medication Class(es)	RxCUI Codes
		541845, 542190, 542195, 567115-567116, 884383, 997006-997007, 1165346-1165347, 1169988-1169989, 1177512-1177513
Lovastatin / niacin	Statin, Niacin	352387, 327008, 379039, 582041-582043, 644112, 729604-729605, 791833, 791836, 791838, 791844, 791846, 1165344-1165345, 1171320, 1171323
Mipomersen	Other non-Statin	1367838-1367848
Niacin	Niacin	7393, 93469, 198024, 198759, 199143, 200015, 205199, 212579, 218728, 219957, 243782, 247747, 260852, 311944, 311951, 311955, 311959-311960, 311963, 314131, 316337-316338, 316341-316344, 316350, 346366, 373053, 380894, 572749-572750, 574313, 575157-575159, 575210, 572749-572750, 647346, 763166-763167, 796544, 848987, 881376, 1007270, 1088776-1088779, 1098134-1098135, 1098141-1098144, 1157448-1157449, 1158120-1158121, 1176605-1176606, 1182877-1182879, 1183356, 1184221-1184222, 1539267, 2001471-2001480, 2001482-2001492, 2121690-2121691
Pitavastatin	Statin	861612, 861634, 861640, 861642-861643, 861645-861646, 861648, 861650, 861652, 861654, 1163274, 1164454, 1179132-1179133, 1944734, 2001252-2001268, 2001322, 2001324, 2001331, 2001333, 2001336, 2001338
Pravastatin	Statin	42463, 203144, 203333, 368322, 373560, 904457-904460, 904466-904469, 904474-904477, 904480-904483, 1161691-1161692, 1184638-1184639
Pravastatin / aspirin	Statin	904664, 904668
Rosuvastatin	Statin	301542, 320864, 323828, 402354, 405880, 859418-859421, 859423-859426, 859746-859753, 1157991-1157992, 1173051, 1173053, 2167555-2167575
Rosuvastatin / ezetimibe	Statin, Ezetimibe	2535744-2535751, 2536055-2536066
Simvastatin	Statin	36567, 104490-104491, 152923, 196503, 198211, 200345, 208220, 213319, 312961-312962, 314231, 316672-316675, 317497, 368276, 373888, 563653-563654, 565109, 568935, 573220, 757701-757705, 1158753-1158754, 1187973-1187974, 1295323, 1790676-1790679, 1944257-1944266
Simvastatin / ezetimibe	Statin, Ezetimibe	476345, 476348-476351, 484211, 495215, 1163791-1163792, 1245416, 1245420, 1255426, 1254430, 1245437-1245441, 1245445, 1245449
Simvastatin / niacin	Statin, Niacin	761906-761907, 761909, 762970, 763226, 763228, 763230, 763232, 763234, 763236, 803516, 999935, 999937-999939, 1158108-1158109, 1182624, 1182627, 1372731

RxCUI = RxNorm concept unique identifier.
