# Supplementary material for: Effect of clinical decision support for severe hypercholesterolemia on low-density lipoprotein cholesterol levels
Source: NPJ Digit Med. 2024 Mar 18;7:73. doi: 10.1038/s41746-024-01069-w (PMC10948900; doi:10.1038/s41746-024-01069-w)
Supplement: Supplementary file 2 — Reporting Summary [file 41746_2024_1069_MOESM2_ESM.pdf]

nature portfolio

Corresponding author(s): Iftikhar J. Kullo

Last updated by author(s): Dec 21, 2023

Reporting Summary

Nature Portfolio wishes to improve the reproducibility of the work that we publish. This form provides structure for consistency and transparency in reporting. For further information on Nature Portfolio policies, see our Editorial Policies and the Editorial Policy Checklist.

Statistics

For all statistical analyses, confirm that the following items are present in the figure legend, table legend, main text, or Methods section.

n/a	Confirmed	
☐	☒	The exact sample size (*n*) for each experimental group/condition, given as a discrete number and unit of measurement
☐	☒	A statement on whether measurements were taken from distinct samples or whether the same sample was measured repeatedly
☐	☒	The statistical test(s) used AND whether they are one- or two-sided<br>*Only common tests should be described solely by name; describe more complex techniques in the Methods section.*
☐	☒	A description of all covariates tested
☐	☒	A description of any assumptions or corrections, such as tests of normality and adjustment for multiple comparisons
☐	☒	A full description of the statistical parameters including central tendency (e.g. means) or other basic estimates (e.g. regression coefficient) AND variation (e.g. standard deviation) or associated estimates of uncertainty (e.g. confidence intervals)
☐	☒	For null hypothesis testing, the test statistic (e.g. *F*, *t*, *r*) with confidence intervals, effect sizes, degrees of freedom and *P* value noted<br>*Give P values as exact values whenever suitable.*
☒	☐	For Bayesian analysis, information on the choice of priors and Markov chain Monte Carlo settings
☒	☐	For hierarchical and complex designs, identification of the appropriate level for tests and full reporting of outcomes
☒	☐	Estimates of effect sizes (e.g. Cohen's *d*, Pearson's *r*), indicating how they were calculated

Our web collection on statistics for biologists contains articles on many of the points above.

Software and code

Policy information about availability of computer code

Data collection	No software was used.
Data analysis	Analyses were performed using SAS software, version 9.4 (SAS Institute, Inc.).

For manuscripts utilizing custom algorithms or software that are central to the research but not yet described in published literature, software must be made available to editors and reviewers. We strongly encourage code deposition in a community repository (e.g. GitHub). See the Nature Portfolio guidelines for submitting code & software for further information.

Data

Policy information about availability of data

All manuscripts must include a data availability statement. This statement should provide the following information, where applicable:

- Accession codes, unique identifiers, or web links for publicly available datasets
- A description of any restrictions on data availability
- For clinical datasets or third party data, please ensure that the statement adheres to our policy

The data used and analysed during the current study are available from the corresponding author on reasonable request.

Research involving human participants, their data, or biological material

Policy information about studies with human participants or human data. See also policy information about sex, gender (identity/presentation), and sexual orientation and race, ethnicity and racism.

Reporting on sex and gender	*Use the terms sex (biological attribute) and gender (shaped by social and cultural circumstances) carefully in order to avoid confusing both terms. Indicate if findings apply to only one sex or gender; describe whether sex and gender were considered in study design; whether sex and/or gender was determined based on self-reporting or assigned and methods used. Provide in the source data disaggregated sex and gender data, where this information has been collected, and if consent has been obtained for sharing of individual-level data; provide overall numbers in this Reporting Summary. Please state if this information has not been collected. Report sex- and gender-based analyses where performed, justify reasons for lack of sex- and gender-based analysis.*
Reporting on race, ethnicity, or other socially relevant groupings	Self-reported race and ethnicity information was obtained from the electronic health record, and are summarized in Table 2. Race was categorized as American Indian or Alaskan Native, Asian, Black or African American, White, other, and unknown. Other race included Native Hawaiian, Pacific Islander, and mixed race. Ethnicity was categorized as Hispanic or Latino, not Hispanic or Latino, and unknown.
Population characteristics	As patients in silent mode and active mode were matched, both groups had similar mean (standard deviation [SD]) age (56.1 (11.8) y vs. 55.9 (11.8) y) with 36.0% males in both modes. The mean (SD) baseline LDL-C level in both groups was similar (211.3 (27.4) mg/dL vs. 209.8 (23.9) mg/dL; P=0.26). Self-reported race was similar in both groups and most patients identified as White (90.0% in silent mode vs. 91.9% in active mode; P=0.81). The proportions of those with hypertension, obesity, and who smoked were also similar between the two groups. The use of lipid lowering therapy (LLT) prior to the alert was similar in the silent and active mode groups due to matching. Only 11.2% of individuals in each group were on LLT within 30 days prior to alert trigger.
Recruitment	We employed a pre-post implementation study design to investigate whether implementation of the CDS for severe hypercholesterolemia/possible FH was associated with lower LDL-C in patients with levels ≥190 mg/dL. We excluded individuals who had declined research participation (64 were excluded from the silent mode group and 57 from the active mode group) and 1 individual who had an erroneous LDL-C result, which was corrected to <190 mg/dL at alert trigger. The CDS alert triggered for 901 patients in silent mode and for 970 patients in active mode. Of these, 836 met study inclusion criteria in the silent mode group and 889 in the active mode group. After matching individuals in silent mode and active mode in a 1:1 ratio based on age (±5 years), sex, and baseline LLT regimen which was defined as any statin use, statin intensity, and number of LLT medication classes, a total of 1600 patients were identified, 800 in the silent mode group and 800 in the active mode group. There were 24 individuals in whom the CDS alert triggered both in silent and active mode periods - they were included in the silent mode period only due to difficulty in the attribution of measured outcomes. Similarly, if an individual had multiple CDS alert triggers within a period, the earliest alert date was used for analyses.
Ethics oversight	This study was conducted as a quality improvement project at Mayo Clinic from August 2018 to January 2023 and was therefore considered exempt by the Mayo Clinic Institutional Review Board.

Note that full information on the approval of the study protocol must also be provided in the manuscript.

Field-specific reporting

Please select the one below that is the best fit for your research. If you are not sure, read the appropriate sections before making your selection.

☒ Life sciences ☐ Behavioural & social sciences ☐ Ecological, evolutionary & environmental sciences

For a reference copy of the document with all sections, see nature.com/documents/nr-reporting-summary-flat.pdf

Life sciences study design

All studies must disclose on these points even when the disclosure is negative.

Sample size	The clinical decision support (CDS) tool was deployed for 3 months in silent mode (08/13/2020-11/12/2023) and for 3 months in active mode (11/13/2020-02/12/2021). The CDS tool was triggered by LDL-C testing with results ≥190 mg/dL. The study population contained all unique patients with CDS triggers during each study period (silent or active). Power calculations were performed using estimated sample sizes for 3 month periods to determine the difference in LDL-C that could be detected.
Data exclusions	Patients were excluded if they did not authorize the use of their medical record for research or if the LDL-C value at the CDS alert date was <190 mg/dL. Patients who had CDS alerts triggered during both the silent and the active alert periods were only included in the silent alert group. Finally, patients the silent alert group were matched to patients in the active alert group on sex, age at alert (+/- 5 years), and lipid lowering medication use. Patients who had no matches found were excluded from the final analysis data.
Replication	A sensitivity analysis was performed for the primary outcome of LDL-C values following the alert. The primary analysis used the earliest LDL-C result in the 1-12 months after the alert, while the sensitivity analysis used the most recent LDL-C result in the 1-12 months after the alert. The results remained similar.
Randomization	This study did not involve randomization. Patients from each study group were matched on covariates of interest (sex, age at alert, and lipid lowering medication use) to balance both groups.

Blinding	Blinding was not applicable to our study.

Reporting for specific materials, systems and methods

We require information from authors about some types of materials, experimental systems and methods used in many studies. Here, indicate whether each material, system or method listed is relevant to your study. If you are not sure if a list item applies to your research, read the appropriate section before selecting a response.

Materials & experimental systems

n/a	Involved in the study
☒	☐ Antibodies
☒	☐ Eukaryotic cell lines
☒	☐ Palaeontology and archaeology
☒	☐ Animals and other organisms
☒	☐ Clinical data
☒	☐ Dual use research of concern
☒	☐ Plants

Methods

n/a	Involved in the study
☒	☐ ChIP-seq
☒	☐ Flow cytometry
☒	☐ MRI-based neuroimaging

Plants

Seed stocks	*Report on the source of all seed stocks or other plant material used. If applicable, state the seed stock centre and catalogue number. If plant specimens were collected from the field, describe the collection location, date and sampling procedures.*
Novel plant genotypes	*Describe the methods by which all novel plant genotypes were produced. This includes those generated by transgenic approaches, gene editing, chemical/radiation-based mutagenesis and hybridization. For transgenic lines, describe the transformation method, the number of independent lines analyzed and the generation upon which experiments were performed. For gene-edited lines, describe the editor used, the endogenous sequence targeted for editing, the targeting guide RNA sequence (if applicable) and how the editor was applied.*
Authentication	*Describe any authentication procedures for each seed stock used or novel genotype generated. Describe any experiments used to assess the effect of a mutation and, where applicable, how potential secondary effects (e.g. second site T-DNA insertions, mosiacism, off-target gene editing) were examined.*
